# Independent Journalism

**Published:** 1877-12

**Authors:** 


### INDEPENDENT JOURNALISM.

In the Canada Journal of Dental Science, for November 1877, is the following language from the pen of the Editor. "It is a fact that our dental journals are mainly advertising mediums for manufacturers and colleges who own them; that while they are in a measure exponents of professional thought, they are first of all monthly advertisments for their proprietors. We find no fault with these gentlemen. It pays. But we do think that a body like the dentist of the United States, out of all the journals they possess, might have at least one as independent of any manufacturer, college or other such business interest as this unpretending Canadian venture. Naturally no advertising medium of a manufacturer, when the bulk of the book is occupied with his own productions, and other manufacturers are deterred by excessive rates from advertising, can or will do justice to the good in other Nazareths. We have plenty of able men, competent to conduct independent journals, quite as well as those conducted by our friends the manufacturers. In no sense would this be a slight; because a profession like dentistry ought to have its independent journals, where impartial examination would be made of every so called 'improvement,' and we would know what to trust and what to reject."

The above pharagraph has been published a month and the affairs of the profession go on about as they did before.

Now really we would be pleased to know what Bro. Beers wants? What does he mean? Is it that the editors of all the devtal journals in the United States are subservient to and are the mere tools of the manufacturers and dealers in dental goods, and of the colleges etc. One would suppose from Dr. B's complaint that no editor of a dental journal dares wink without consulting his master!

His entire desire for independent journalism is not confined to the editor of the C. J. D. S., we hear it often and from various quarters; so that he need not imagine that he has struck a new lead on that "claim," he is simply digging in the old hole. His wail for independent journals has become chronic, and that too after several have starved to death while trying to be independant,—yes too independent to live.

Now what do all these worded complaints mean by independent journalism? Do they mean that the editors of our dental journals choose and prepare the matter, either original communications, selections or editorials, under the dictation or direction of manufacturers, dealers, or colleges? If so, then we do not hesitate to say positively that the *facts* do not sustain the charge. Or do they mean that the editors of our journals are in the habit of writing commendatory of worthless instruments, appliances or modes of practice. If that is the charge, it would be well to specify, point out the criminal, and don't shoot the whole tribe.

Or would they have the journals converted into a cat-o-nine-tails, with which to lash through the world, any luckless manufacturer or dealer in dental goods, who, in his enthusiasm, might chance to place a higher value upon his wares than others might do.

Now we would simply suggest that as the C. J. D. S. is the only independant dental journal on this continent that it attend to the flagellation business: and the few journals that are left will attend to the balance of the work. It is wonderful what a division of labor will accomplish.

Oh! in the next pharagraph it is written; "We have been turning over the advertising pages of many of these periodicals, extending back many years. It is a suggestive fact that a long array of exhaustive puffs and extravagant statements made to the profession, present themselves about articles which were never worthy of honest consideration, and of many which were arrant impositions. Every new article to-day is boosted into notice in the same way."

Oh, now we begin to see it is the advertising sheets that are stitched up with the journals that Bro. B. is fighting, while in that contest we are spectators.

The *editors* have nothing to do with the advertising pages, and are in no sense responsible for them. This is true of some of our dental journals if not all.

Are the editors of our dental journals dolts who can be led around by the nose by every and any one who may chance to manufacture, or deal in dental goods?

Does the mere fact of stitching with and printing with in the same cover with a dental journal, fifteen, thirty, or forty pages of advertisements, render the former worthless or of less value? If so how and why?

Is the tenth edition of Harris' Principles and Practice of Dental Surgery, published by Lindsay & Blakiston of less value because there are forty pages of the publisher's advertisements bound with the volume?

Is Wagner's Manual of general pathology published by Wm. Wood, & Co., greatly deteriorated because there are forty pages of the publisher's advertisements, at the end of the volume? And are the authors of these and many other books that might be mentioned not independant, but the servants to do the bidding of the publishers and advertisers.

Away with all this clatter and nonsense about the absence of independent journalism, in the dental profession. Dentists write about as freely and independantly as any other class of men, and they have no difficulty in finding journals to publish their communications when they are of value or interest, and in condition to be published.

